# Design and Operational Elements of the Robotic Subsystem for the e.deorbit Debris Removal Mission

**DOI:** 10.3389/frobt.2018.00100

**Published:** 2018-08-31

**Authors:** Steffen Jaekel, Roberto Lampariello, Wolfgang Rackl, Marco De Stefano, Nassir Oumer, Alessandro M. Giordano, Oliver Porges, Markus Pietras, Bernhard Brunner, John Ratti, Quirin Muehlbauer, Markus Thiel, Stephane Estable, Robin Biesbroek, Alin Albu-Schaeffer

**Affiliations:** ^1^German Aerospace Center (DLR), Institute of Robotics and Mechatronics, Wessling, Germany; ^2^Department of Informatics, Technical University of Munich, Garching, Germany; ^3^Munich University of Applied Sciences, Munich, Germany; ^4^OHB System AG, Wessling, Germany; ^5^MDA Corporation, Brampton, ON, Canada; ^6^AIRBUS DS, Bremen, Germany; ^7^European Space Research and Technology Centre, Noordwijk, Netherlands

**Keywords:** on-orbit servicing, active debris removal, space robot, visual tracking, robot gripper, clamping mechanism, e.deorbit

## Abstract

This paper presents a robotic capture concept that was developed as part of the e.deorbit study by ESA. The defective and tumbling satellite ENVISAT was chosen as a potential target to be captured, stabilized, and subsequently de-orbited in a controlled manner. A robotic capture concept was developed that is based on a chaser satellite equipped with a seven degrees-of-freedom dexterous robotic manipulator, holding a dedicated linear two-bracket gripper. The satellite is also equipped with a clamping mechanism for achieving a stiff fixation with the grasped target, following their combined satellite-stack de-tumbling and prior to the execution of the de-orbit maneuver. Driving elements of the robotic design, operations and control are described and analyzed. These include pre and post-capture operations, the task-specific kinematics of the manipulator, the intrinsic mechanical arm flexibility and its effect on the arm's positioning accuracy, visual tracking, as well as the interaction between the manipulator controller and that of the chaser satellite. The kinematics analysis yielded robust reachability of the grasp point. The effects of intrinsic arm flexibility turned out to be noticeable but also effectively scalable through robot joint speed adaption throughout the maneuvers. During most of the critical robot arm operations, the internal robot joint torques are shown to be within the design limits. These limits are only reached for a limiting scenario of tumbling motion of ENVISAT, consisting of an initial pure spin of 5 deg/s about its unstable intermediate axis of inertia. The computer vision performance was found to be satisfactory with respect to positioning accuracy requirements. Further developments are necessary and are being pursued to meet the stringent mission-related robustness requirements. Overall, the analyses conducted in this study showed that the capture and de-orbiting of ENVISAT using the proposed robotic concept is feasible with respect to relevant mission requirements and for most of the operational scenarios considered. Future work aims at developing a combined chaser-robot system controller. This will include a visual servo to minimize the positioning errors during the contact phases of the mission (grasping and clamping). Further validation of the visual tracking in orbital lighting conditions will be pursued.

## 1. Introduction

Due to the high amount of satellites that have been brought into orbit in the past decades, the space environment around the Earth has been heavily cluttered with debris that is becoming an increasing endangerment for current and future space missions. Collisions between orbiting elements result in a cloud of space debris, potentially leading to a chain reaction (Kessler syndrome) that may finally render the low and geostationary orbits non-operational (Liou, 2011). In addition, the uncontrolled de-orbiting of large space debris that does not burn up completely during re-entry constitute an increased risk for the highly populated Earth surface and a currently unresolved legal issue. In the given e.deorbit scenario, a chaser satellite that features a robotic arm captures, stabilizes and de-orbits a target satellite. The target was defined to be ENVISAT, an eight-ton formerly Earth-observing satellite, that is defective and tumbling uncontrolled with a constantly declining spin rate of currently about 3 deg/s. However, due to potential future orbital collisions or internal incidents, such as uncontrolled valve venting, the current study considers up to 5 deg/s around any axis. An overview of the whole e.deorbit mission, including a detailed target description, as well as multiple capture and de-orbit options, e.g., with a flexible net, can be found in Wieser et al. (2015). This paper concentrates on the robotic capture solution, its system design and envisaged operations. It outlines the findings gained and analyses conducted until phase B1 within two independent studies, with industrial cooperation with OHB Systems and Airbus DS, respectively.

This paper is structured as follows: After a short description of the state of the art of related orbital robotic systems, the robotic operational strategy for performing the e.deorbit mission is described. Following that, descriptions of the design of relevant hardware elements of the robotic system are given. This includes the robotic joints, gripper and clamping mechanism. Subsequently, results of kinematic and dynamic simulations are presented, aiming to prove the feasibility of the mission with the proposed technology and methods. These include analyses of the robot manipulator kinematics, the robot link flexibility dynamics, as well as the robot joint internal loads during some of the critical mission phases. Following, a performance analysis of defining elements in the control system is given. This firstly includes the visual tracking, described through a Monte Carlo analysis, and secondly, the interaction between the chaser and the robotic manipulator controllers, realized through a coupled architecture approach. Finally, the conclusions and future work are outlined.

## 2. State of the art

**Figure 1:**
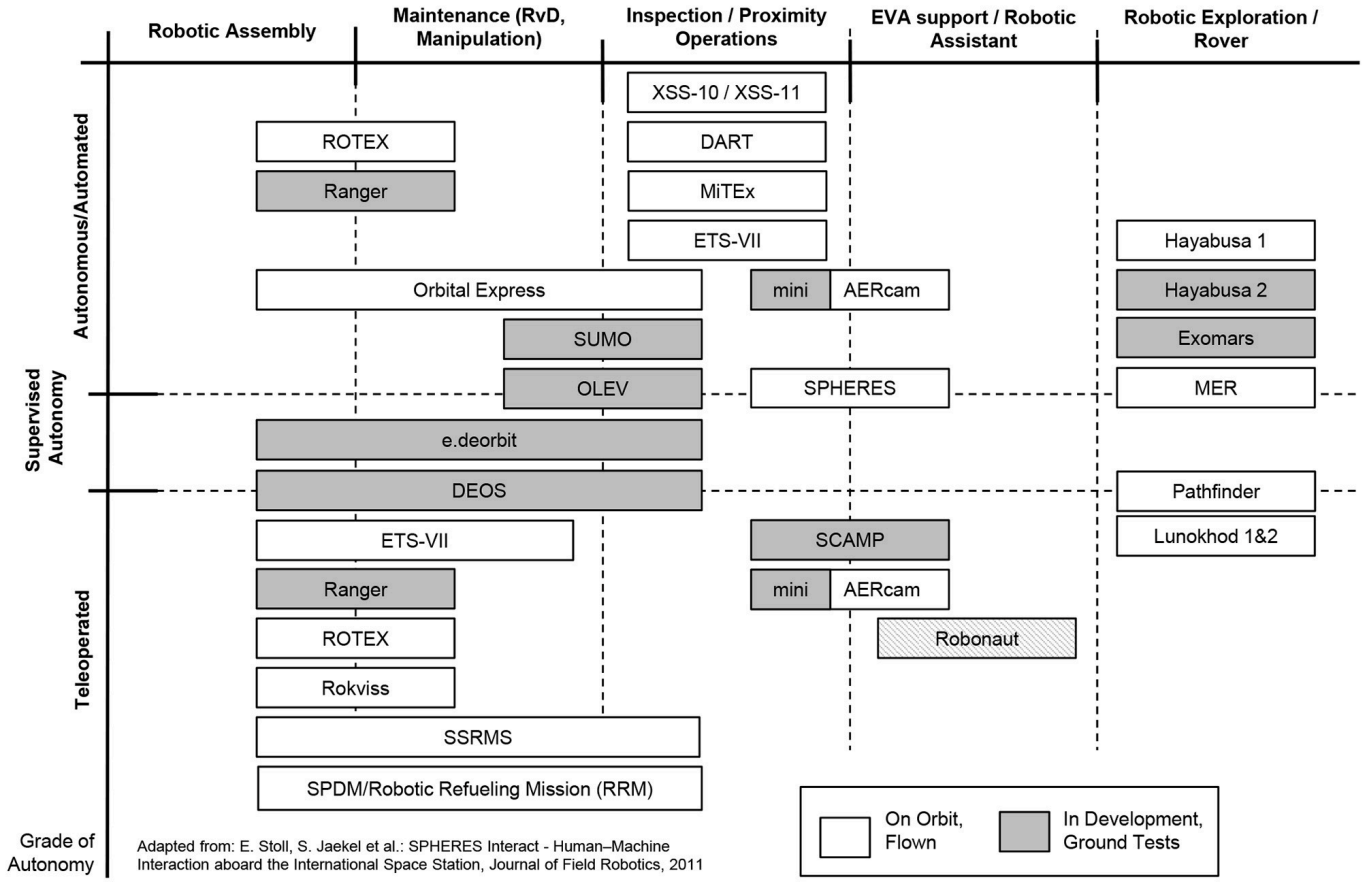
Overview and classification of missions displaying capabilities for robotic on-orbit servicing and active debris removal. The missions are classified in different tasks: assembly, maintenance, inspection, assistance and exploration, as well as autonomous capabilities: teleoperated, supervized, autonomous.

Apart from a controlled capture and de-orbiting, as planned within the e.deorbit scenario, the described robotic concept can also be used for on-orbit servicing (OOS) tasks, i.e., extending the lifespan of operational satellites through refueling or by repairing and replacing specific elements of a non-operational satellite. Utilizing space robotics for active debris removal (ADR) and servicing in orbit is a very promising approach as there have been multiple missions and investigations in the past to strengthen this line of technology, cf. Figure 1. There are four major groups of robotic applications in space that can be defined. Using the Shuttle and Space Station Robotic Manipulator System (SRMS, SSRMS), respectively, the International Space Station (ISS) was assembled out of several modules by applying the principle of in-space robotic assembly (ISRA) (Mohan and Miller, 2009). Small robotic satellites are planned to serve for inspection purposes (Stoll et al., 2012) and NASA's Robonaut (Diftler et al., 2012) or comparable systems such as DLR's humanoid robot Justin (Zacharias et al., 2010) are candidates for future extra-vehicular activity (EVA) support operations. Similar to ISRA and EVA support, dexterous robotic manipulators are planned to be utilized to capture, maintain and/or de-orbit operational and defective satellites within on-orbit servicing and active debris removal missions (Hirzinger et al., 2004). Finally, robotic exploration of other celestial bodies, such as the Moon, Near Earth Objects (NEOs), or Mars is envisaged or has already been accomplished (Biesiadecki and Maimone, 2006).

Currently, the deployment of regularly used robotic systems in space is limited to the Space Station Remote Manipulator Systems (SSRMS) (Aikenhead et al., 1983), the Japanese Experiment Module Remote Manipulator System (JEM-RMS) (Matsueda et al., 1991), and the Mobile Servicing System (MBS) (Werstiuk and Gossain, 1987) aboard the ISS. The MBS also features a Special Purpose Dexterous Manipulator (SPDM) (Mukherji et al., 2001) that recently conducted the Robotic Refueling Mission (RRM) demonstrating remotely controlled robotic servicing, including refueling with a dedicated experimental platform aboard the ISS (Cepollina and Reed, 2017). These systems can also be teleoperated by the crew and are being used for extravehicular activity (EVA) support, space station assembly and vehicle docking. The Shuttle Remote Manipulator System (SRMS) was also used for satellite repair operations (Hubble). In combination with the SRMS, the Orbiter Boom Sensor System (OBSS) (Greaves et al., 2005) was utilized for the inspection of the Shuttle's heat protection tiles.

In addition to the robotic servicing capabilities that are bound to the now decommissioned Shuttle or to the ISS, several satellite-based demonstrators were flown in orbit to demonstrate the possibility of on-orbit servicing. The most important demonstrators and missions are the Robot Technology Experiment (ROTEX) (Hirzinger et al., 1993), developed by the German Aerospace Center (DLR), the Ranger telerobotic flight experiment (RTFX) from the University of Maryland (Roderick et al., 2004), the Japanese Engineering Test Satellite VII (ETS-VII) (Oda et al., 1996; Yoshida, 2003), the German Robotic Component Verification experiment aboard the ISS (ROKVISS) (Albu-Schaffer et al., 2006), the Demonstration of Autonomous Rendezvous Technology (DART) (Howard et al., 2004) by NASA, the Experimental Small Satellite-10 (Davis and Melanson, 2004) and -11 (Madison, 2000) (XSS-10/11), the Micro-Satellite Technology Experiment's (MiTEx) (Osborn et al., 2007), the Orbital Express (Shoemaker and Wright, 2003) mission by DARPA, as well as the German Orbital Servicing Mission study (DEOS) (Sellmlaier et al., 2011). A comprehensive overview of the above-named missions and experiments can be found in Flores-Abad et al. (2014). The DEOS project, carried out by the DLR, investigated technologies to perform satellite rendezvous and close proximity operations, as well as to capture a tumbling and uncooperative target satellite with a dexterous manipulator in autonomous or in teleoperation mode. Although this particular project did not continue beyond the preliminary design phase, the work on the robotic capture system is still ongoing, and was used as the technological heritage for the e.deorbit mission study.

## 3. Operational strategy for capture and de-orbiting

After a careful analysis of the target structure and of the related operational challenges, the grasping point was chosen to be on the Launch Adapter Ring (LAR). This provides a very solid, stiff, well-exposed, and well-defined structure, which is required for sufficiently designing a capable gripper that is able to achieve a stable form and force closure with it. Any other exposed structures like the big synthetic aperture antenna (SAR), antenna and solar array booms have been quickly ruled out due to the before mentioned criteria. The robotic operations then consist of two major tasks: firstly, the grasping of the LAR by means of the robot manipulator from some predefined position of the chaser relative to the target, and secondly, the subsequent positioning of the chaser onto the LAR, to allow for the closure of a firm connection between the two spacecraft through a dedicated clamping mechanism, for the subsequent de-orbiting maneuver.

**Figure 2:**
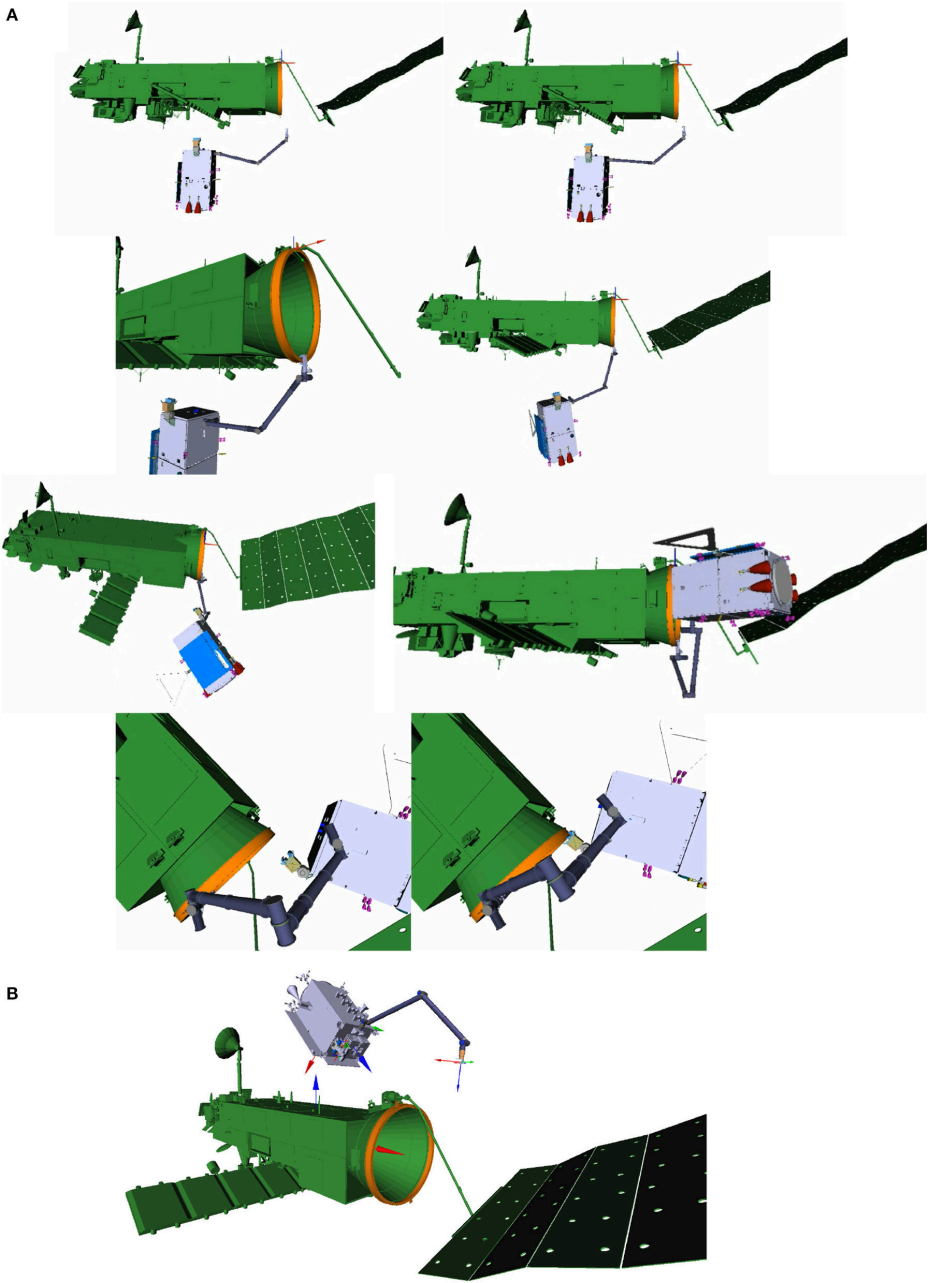
Chaser satellite in synchronized flight at the arm delivery point with the robot arm shown in its initial configuration, respectively, for the bottom **(A)** and top **(B)** approach. **(B)** Also indicates the main coordinates frames in the spacecraft CoGs with the colors rgb = xyz and the target's x-axis being congruent to the LAR symmetrical axis. The capture phases shown in A are from top to bottom: arm approach, capture with gripper, transition into clamping position, and finally, clamping.

Within the two studies, two approach solutions have been identified. Figure 2, **Top** depicts several phases of the capture operation in the OHB scenario, in which the chaser approaches from the bottom (-z) side where most of ENVISAT's instruments including the large synthetic aperture radar antenna are located. Figure 2, **Bottom** depicts the AIRBUS approach with the chaser approaching from the top (+z) side, it also identifies the main body frames of chaser and target, respectively. The main advantage of the bottom approach is the absence of umbilicals at this side of the ring, such as the antenna boom motor and connectors that were previously used to connect ENVISAT with the launcher vehicle, giving greater robustness for the grasp maneuver. This comes at the cost of less distance between the outer spacecraft structures, due to a shift in +z between the center of the adapter ring structure and the horizontal symmetrical z-plane of the cuboid main body. With respect to kinematic reachability of the robotic manipulator for capture and transition, both approaches have been identified to be feasible. However, the large solar array, the exact position of which is uncertain to a specific degree, poses less of a risk and hence, increases flexibility, when approaching from the top side.

The tumbling motion of the target was defined by ESA to be up to 5*deg*/*s* about any axis. Figure 2, **Top** depicts the chaser in a synchronized flight with the spinning target, at the arm delivery point, where it is aligned with the target's center of mass and its maximal principal axis of inertia, at a distance of approximately 4m. The previous rendezvous maneuver of the chaser, described in detail in Wieser et al. (2015), comprises an approach along the major axis of rotation, starting from an inspection point at some 50 m distance, and with continuous and iterative synchronization of the relative motion and attitude. However, the internal joint torques in the robotic manipulator can be minimized by choosing the approach from the top, as shown in Figure 2, **Bottom**, where the distance between the chaser's center of mass and the grasping point on the LAR is smaller, see section 5.3.

During the synchronization maneuver, the robotic arm remains in the stowed configuration. When the chaser arrives at the arm delivery point, the robotic arm is unfolded and brought into a predefined initial configuration for the following grasping phase. A pre-planned robotic arm approach trajectory is executed toward a preselected grasping point on the target. The approach trajectory is planned on ground, with the motion planning method described in Lampariello and Hirzinger (2013), however with the additional condition that the chaser is regarded to be fixed with respect to the target body frame. The guidance, navigation and control (GNC) subsystem of the chaser, which holds the chaser synchronized to the target, is fed with the platform-mounted LIDAR sensor measurements, being used for relative pose estimation. The resulting robot arm trajectory, which guarantees feasibility with respect to motion constraints, such as singularity avoidance and end-effector camera field-of-view requirements, is stored on board the chaser for online execution.

As the robot controller moves the robotic arm toward the predefined grasping point on the LAR, internal forces and torques are applied onto the chaser. Section 6.2 analyzes the interdependencies of such a coupled control approach in more detail. In addition, relative positioning and synchronization of the two spacecraft can only be done within the accuracy of the GNC (uncertainty box). Due to these uncertainties, during the capture, some unknown dislocation and residual motion between the two spacecraft can be expected, which the path planner for the arm approach cannot account for. In order to tackle this potential dislocation and drift, the arm-mounted stereo camera system is utilized for closed-loop pose error corrections through visual servoing. Using model-based visual tracking (Panin, 2011) and visual servoing, the manipulator is guided to the grasping point on the LAR, compensating for the unknown relative positioning errors. While the LAR structure is being grasped by the robotic arm, the GNC of the platform is active. After achieving form and force closure with the gripper, the GNC is switched off and residual motion between the two spacecraft is actively damped out using the force-sensitive impedance control of the robot arm. The two satellites are now rigidly connected through the robot arm and rotate further in a free-tumbling motion. After actively de-tumbling the satellite stack using the chaser thrusters, the chaser satellite is brought into a clamping position at the LAR of ENVISAT. By closing the clamping mechanism, a sufficiently stiff connection between chaser and target is realized as a prerequisite for the subsequent stack de-orbit maneuver.

## 4. Robotic components

This section presents the design of the robotic arm, its kinematic setup and internal mechatronic joint composition. Subsequently, the gripper and clamping mechanism designs to achieve form and force closure with the LAR are laid out.

### 4.1. General arm design

**Figure 3:**
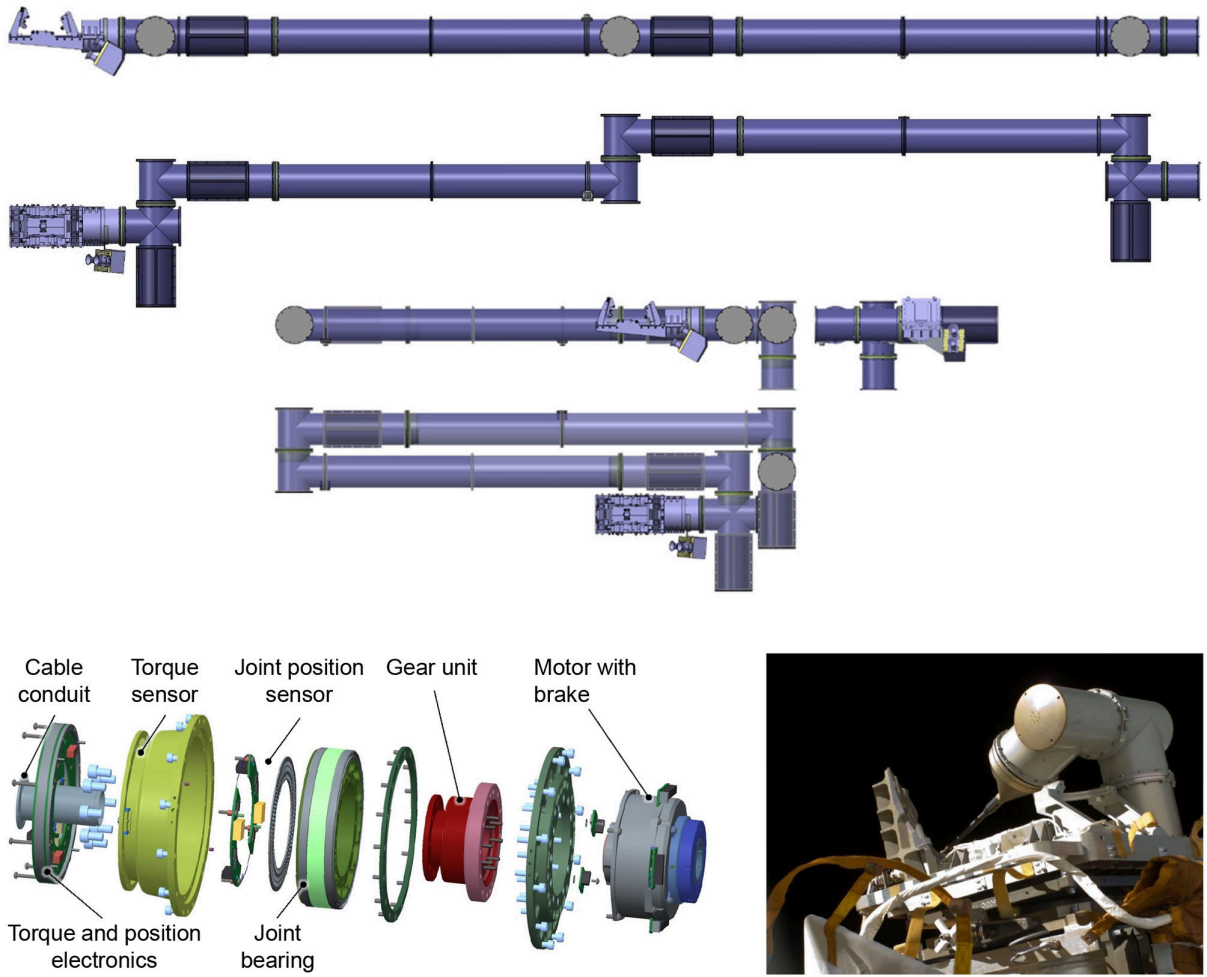
Robotic arm in stretched and stowed configurations with gripper and stereo camera system attached **(Top)**. Explosion view of the integrated joint design for the robotic manipulator **(Bottom, Left)** and ROKVISS experiment with heritage joint design outside the Zvezda service module aboard the ISS **(Bottom, Right)**.

The robotic arm for capturing the designated target, shown in Figure 3, **Top**, has a stretched length of 4.3 m and features seven degrees of freedom (DoF), due to its seven identical revolute joints. This chosen kinematical structure is redundant (of redundancy degree one) with respect to the grasping task, to allow for a greater arm dexterity and robustness to singularities. The kinematic dh parameters are presented in Table 1. The joints are connected by aluminum cylindrical tubes which, together with four additional redundant electronic blocks integrated into the arm assembly, provide the housing for the joint sensors, wiring and electronics. The last block controls the seventh joint as well as the gripper which is built upon a commutable mechatronic design. A linear-driven gripper was designed to achieve full form and force closure of the standardized LAR for Ariane 5. The stereo camera system and illumination are mounted on a special bracket, placed above the seventh joint. During launch and in the early orbit phase, the arm is held down by Frangi-bolts to the chaser platform in the stowed configuration, as shown in Figure 3, **Top**. With the exception of the mechanical structure and hardware mechanisms within the manipulator, the whole actuation string is redundant (one failure tolerant). This includes processing equipment, electronics, cabling, motor windings and sensors.

**Table 1:** Denavit-Hartenberg (DH) parameters of the robotic arm.

a [mm]	0	0	0	0	0	0	0
α [mm]	0	–90	90	–90	90	90	–90
θ [deg]	0	0	180	0	180	0	0
d [mm]	256	168	1900	168	1730	168	420

### 4.2. Joint design

The joint design is based on the heritage from the third generation of the Light-Weight Robot technology (Hirzinger et al., 2002) and from the ROKVISS mission (Albu-Schaffer et al., 2006), depicted in Figure 3, **Bottom, Right**. In the latter mission, two robot joints were space qualified within a five year operation outside the Russian service module of the ISS. The result is a highly integrated and space-qualifiable joint design, following ECSS specifications. Each joint, shown in Figure 3, **Bottom, Left**, features integrated position as well as torque sensors, operating in classical position or active impedance control modes. The latter allows minimizing the possibly detrimental effect of collisions with the target during the grasping phase. For safety purposes, the maximum operational speed of each joint is set to 10 deg/s. The Harmonic Drive gears limit the joint torques to ±80 Nm during nominal operations. The momentary peak torque is 176 Nm. The still ongoing development of these joints aims at meeting the requirements of a broad range of future on-orbit servicing missions.

### 4.3. Gripper design

**Figure 4:**
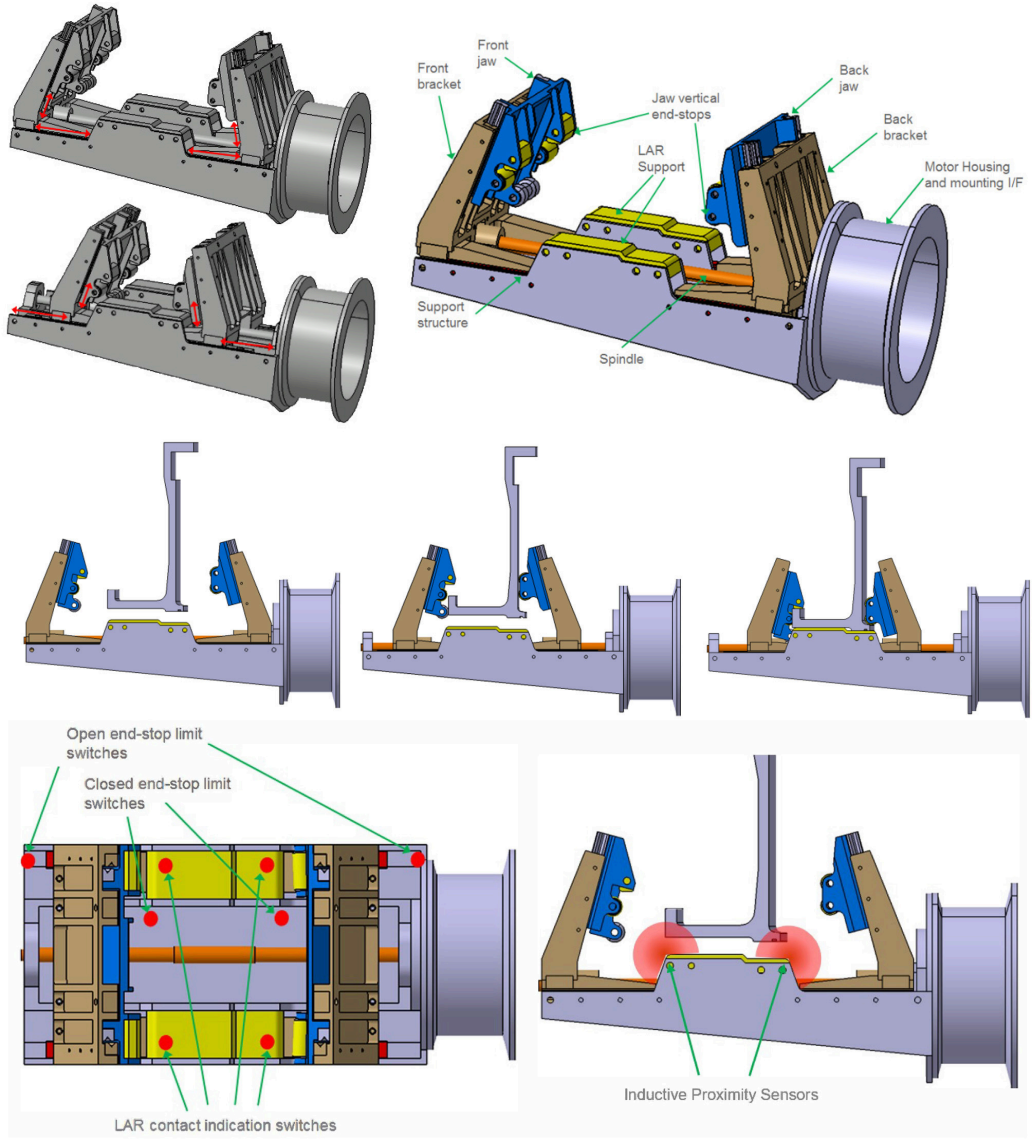
Gripper movable parts **(Top Left)**, overview of general design **(Top Right)** and Clamping process illustration **(Middle)**. Gripper sensor concept with limit switches **(Bottom, Left)** and proximity sensor concept **(Bottom, Right)**.

The gripper design was developed by OHB and is oriented toward the LAR geometry and mechanical properties which are of type ACU 2624. Due to the requirement of grasping the LAR from the outside, and it having a cylindrical, foil-covered surface with only a small extrusion (less than 4 mm in thickness) for vertical fixation, a classical hinge-like approach was found to be inconvenient to achieve the desired 6-DoF force closure. Consequently, the gripper was designed to clamp the LAR using two brackets with a horizontal linear DoF in the radial LAR direction. From a nominal clamping position, the closing of the brackets is initiated and the brackets start moving toward the LAR from both sides. Each bracket is equipped with a jaw featuring an inclined translational DoF. The general design and the degrees of freedom of the movable parts (horizontal clamping bracket and inclined vertical jaw) are illustrated in Figure 4, **Top**. All contact points to the LAR on the gripper side are implemented with roller elements. As a consequence, rolling occurs only for relative motions during capture, centering and clamping. Any sliding contacts between the LAR and the gripper are avoided by design. Successful gripping was verified by contact dynamics simulation on MSC Adams. The simulation further confirmed the anticipated initial grasping position tolerances of 20*mm* lateral and 5*deg* angular. The overall clamping process, including a passive pulldown of the jaws, is illustrated in Figure 4, **Middle**.

A trades study was performed for identifying a convenient sensor suite for internal closed-loop control and status indication (see also Figure 4) (bottom): (1) Micro switches: Micro switches are used for three different functions. For positive LAR capturing indication, four micro switches are located below the LAR support and are triggered when the local interface force is above the threshold. Four positive signals indicate an equal contact pressure of the LAR bottom side to the gripper, application of the pre-defined gripping preload and an equal distribution of the clamping force. Open and closed configuration limit switches indicate that the gripper is in nominal open or closed configuration and avoid running into the hard end-stops. This limit switch is used to turn off motor power when opening and closing the gripper. (2) Motor torque tensor: The torque sensor is used for applying the predefined nominal holding force to the LAR. The force is measured indirectly by the torque sensor and transformed by the previously characterized spindle and ball nut efficiency. (3) Proximity sensors: Light curtain sensors can be applied in order to verify that the LAR lower edge is located within the alignment tolerance envelope. As design alternative to position sensors (1) and proximity sensors (3), an inductive sensor suite is still under consideration due to then additionally available proximity information and contactless operation. An advanced alternative to the light curtain is the usage of inductive sensors at the LAR support. These sensor types provide proximity information at several distributed locations and would allow verifying the correct positioning an alignment of the LAR throughout the gripping operation. However, this sensor type comes at the cost of increased complexity and reduced space heritage.

### 4.4. Clamping mechanism

Once the chaser has successfully captured the target, a rigid link must be established between the two spacecraft to be able to sustain the de-tumbling and de-orbit maneuver loads. This is done by means of a clamping subsystem, located on the chaser's top deck. This subsystem was designed by MDA, and provides two primary functions for the mission: (1) Secure ENVISAT to the chaser by grasping onto a segment of the LAR, and provide structural strength and rigidity during chaser maneuvering and de-orbit engine firings. (2) Adjust the relative orientation of the chaser with respect to ENVISAT to support the alignment of the chaser's main engine thrust vector through the combined stack CoG of the two spacecraft.

During the initial mission operations development, a key consideration was where to clamp onto the spacecraft once it had been captured by the robotic arm. This location needed to be physically well defined, which would allow a clamping system to be designed, and it had to be accessible so that the clamping mechanism could be positioned for capture. As well, it needed to be strong and stiff enough to provide a controllable stack of spacecraft for maneuvering and de-orbiting. After a survey of the Envisat design, the final candidates were the LAR and the solar array launch restraints on the satellite body. Additionally, it was considered to design a large clamp that would grab the Envisat body across its entire width, effectively hugging the satellite. This last option was eliminated as risky, primarily because the grasp would not be deterministic, meaning that the exact point of grasping could vary, and the load capability of the Envisat structure in this application was not known.

Amongst the two remaining options, the LAR was selected because it is rigid, strong, and its geometry is well known. All of these make it an ideal interface for the clamping mechanism. It is also easily accessible at the bottom of ENVISAT. The solar array launch restraints could also have worked due to their exposed location on ENVISAT, but it was decided that the LAR ring provided a more exposed interface for the majority of satellites, therefore making the LAR clamping system solution more commercially attractive for satellite servicing missions.

Once the LAR had been selected as the clamping interface, two key design trades in the design of the clamping mechanism consisted of: (1) The size of the capture envelope: the size and mass of the clamp, vs. the performance of the robotic arm to position the LAR ring within the jaws. (2) The stiffness of the clamped interface: the arc length of LAR ring to clamp onto, minimizing mass, volume and power vs. providing a sufficiently stiff coupling between the two spacecraft to allow for good control over attitude control and de-orbit maneuvers.

For trade (1), an integrated performance analysis of the robotic system after ENVISAT capture was performed, to determine the achievable arm tip positioning accuracy, and therefore LAR positioning accuracy, for a blind autonomous capture, using only proximity sensors to detect the LAR in the capture box. This approach eliminates the need for cameras imaging the clamping system and does not require teleoperation. A capture envelope of +/−21mm in all axes, +/−2deg about any axis was easily achievable in the clamping system design, and allows current robotic arm technology to be used. The goal is to strike a balance so as to minimize the development cost of each element of the system.

For trade (2), a structural analysis was performed of the combined LAR, clamping mechanism and alignment mechanism. It was determined that by far the least stiff element was the LAR itself, and therefore that a longer clamping arc equated to a stiffer system, as the clamping mechanism essentially acts as a local doubler. An arc length of 300*mm* with a clamping preload of 10, 000*N* was selected for e.deorbit, which has been shown through spacecraft ACS performance modeling to result in adequate controllability of the stack, without unnecessary clamping system mass.

The final design of the clamping mechanism consists of a set of motor-actuated parallel jaws with passively compliant clamping fingers and rollers that conform to the profile of the LAR ring as they close on the structure, creating a very stiff connection between the two spacecraft. After successful capture of the target with the robotic arm, the chaser is moved into position by the arm such that the LAR is brought within the jaws. A camera allows for operator verification of the position of the LAR. A pair of photo-interrupt sensors in the clamp build a light curtain to allow the detection of when the robotic arm has aligned the LAR within the capture envelope of the clamp. When tripped, these sensors initiate an autonomous operation to close the jaws onto the LAR ring.

**Figure 5:**
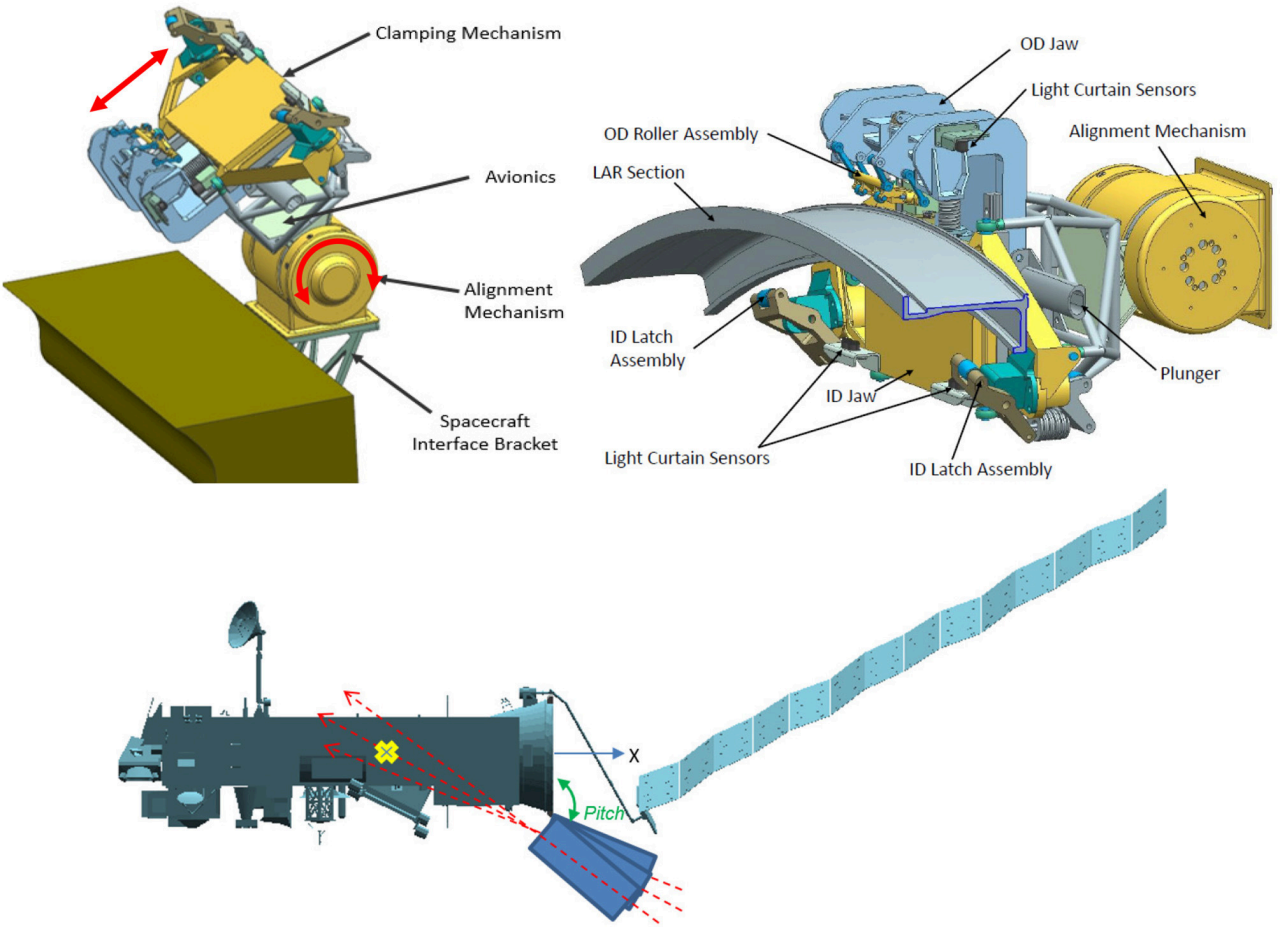
Clamping subsystem configuration **(Top, Left)** and components **(Top, Right)**. Alignment mechanism pitch angle adjustment to shift thrust vector along ENVISAT x-axis **(Middle)**. Comparison between width of the clamping mechanism and occuring deflections during steady state and peak conditions.

At the base of the clamping subsystem is an alignment mechanism, consisting of a rotary joint that can pitch the clamping mechanism relative to the chaser axis through approximately 120*deg*. In addition to a redundant motor, geartrain and brake, the mechanism also incorporates a 16*bit* output rotary position sensor that allows for precision sensing of the absolute position and allows position control to an accuracy of 0.05*deg*. This mechanism is initially used to deploy the system from its stowed launch configuration. Subsequently, it acts as the rotary pitch degree of freedom between the two spacecraft in clamped configuration, and is used to align the chaser thrust vector with the combined stack CoG, as shown in Figure 5. This is a key feature for achieving an accurate de-orbit burn. Since ENVISAT's CoM position is not exactly known, the ability to adjust the pitch angle between the two spacecraft allows for thrust vector corrections as required. Due to the clamping subsystem securing along the circumference of the ENVISAT's LAR, the thrust vector naturally intersects ENVISAT's center line in the y-z plane.

## 5. Kinematic and dynamic simulations and analyses

### 5.1. Arm kinematics

**Figure 6:**
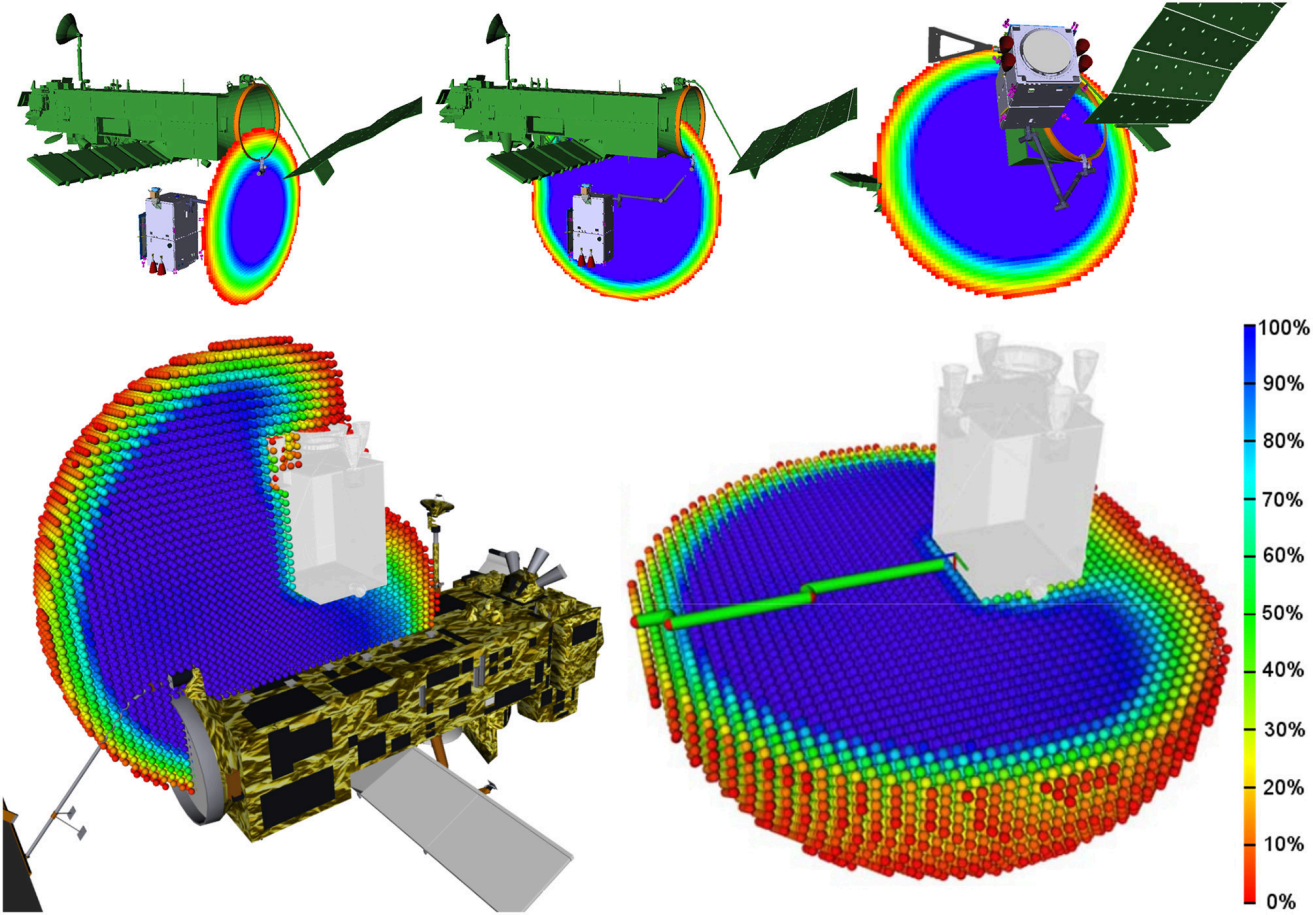
Capability map cross-sections of satellite-mounted manipulator with chaser in arm delivery point for the bottom **(Top Left and Top Middle)** and top **(Bottom, left)** approach, as well as in clamping position **(Top Right)**. Capability maps generated considering robot self-collision and collisions with the chaser are depicted on the bottom.

In order to verify the task-specific performance of the chosen manipulator length and configuration, the kinematics of the manipulator were validated and analyzed using the method of the reachability map (Porges et al., 2014). The reachability map is a hierarchically discretized robot workspace model. The end-effector pose space (*SE*(3)) is discretized into voxels (3D translation) where each voxel has an inscribed structure discretizing the pointing orientation and rotation around it (3D orientation), thus discretizing all six dimensions of the end-effector pose. Such a model provides a global overview of the workspace and the capabilities of the robotic arm in positioning and orienting the end-effector. A kinematic performance measure called reachability index has been derived from the workspace representation as a percentage of reachable rotations within each voxel. In other words, the reachability index reflects what portion of *SO*(3) space is reachable within a small volume of *R*^3^ space. Visualizing all information stored in the reachability map is not feasible. The reachability index encodes the orientation coverage within a small volume of the workspace which makes it possible to visualize the workspace as a capability map, pictured in Figure 6. Each sphere represents a voxel while the color encodes the value of the reachability index in HSV color scale. The workspace models are generated accounting for self-collisions of the robot and collisions of the robot with the chaser body. This is important to make sure that the workspace model properly reflects the overall system capabilities.

Validation is performed by querying the reachability map for existence of the target end-effector pose, and maximizing the reachability index which in turn maximizes the ability of the robot to rotate the end-effector. Figure 6, **Top** shows three cross-sections of the capability map of the satellite-mounted manipulator with the chaser in the arm delivery point and in the clamping position. The scale (bottom right) indicates the ratio of discretized end-effector orientations that are reachable. Within the dark blue area, the end-effector has an optimal manipulability for grasping from any direction. Green volumes indicates feasible and red undesired reachability index values. The bottom row of Figure 6 depicts the top approach arm delivery point pose (left) and the capability map cross-section with the collision model of the robot (right). In this example, the visualized workspaces were generated with collisions in consideration. Reachability maps can be applied beyond the scope of analysis, for example, an on-line determination of robot base pose while performing a grasping operation. Such methods are described in Vahrenkamp et al. (2013). Methods of mitigating single joint failure based on the reachability map workspace model are currently under review.

### 5.2. Arm link flexibility

**Figure 7:**
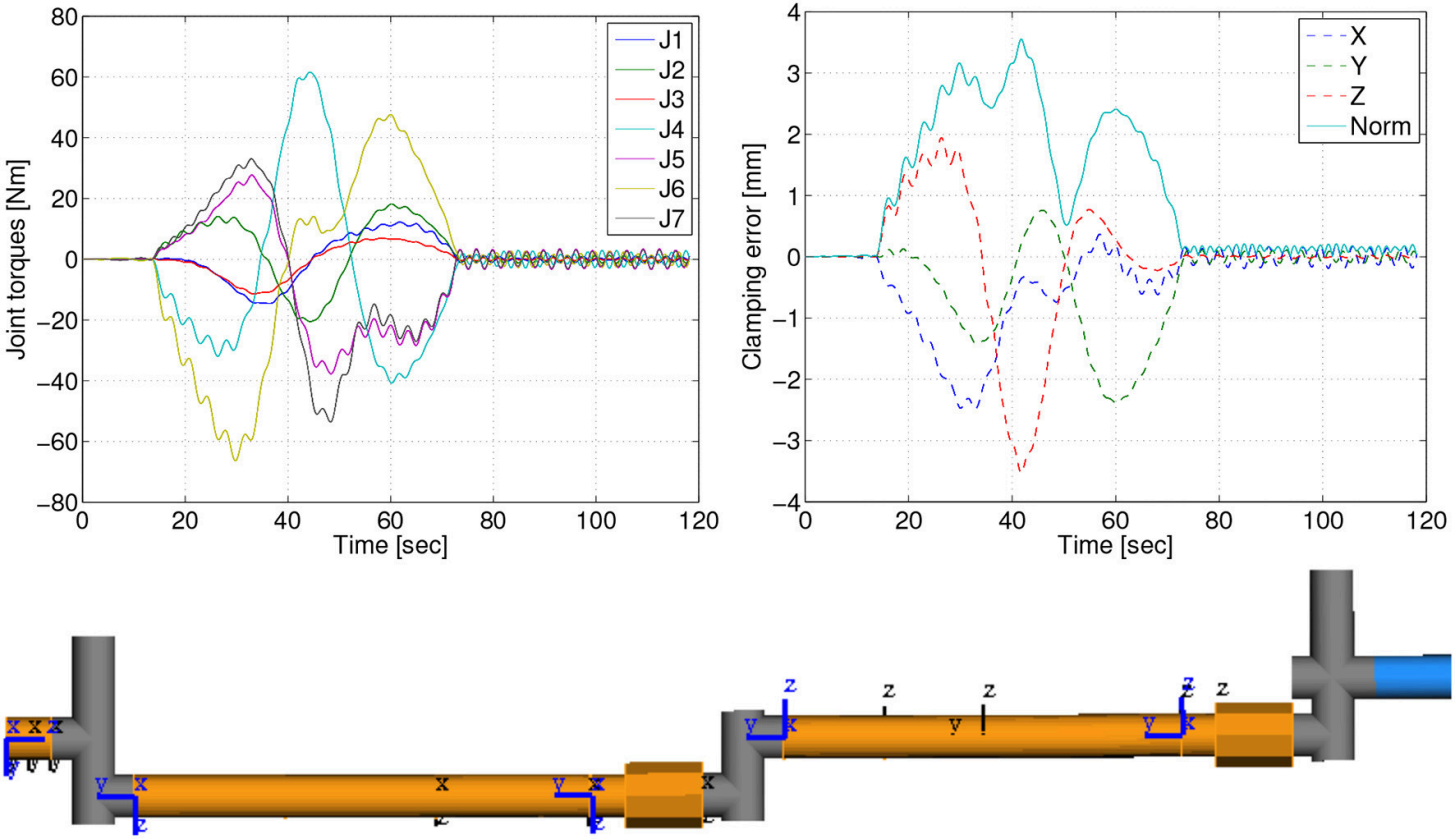
Effects of the link flexibility for a 60s transitioning maneuver: joint torques **(Top, Left)**, positioning error of the clamping interface due to flexible displacements **(Top, Right)** and overview of modeled arm elements **(Bottom)**: flexible links (orange), rigid links (gray), rigid gripper (blue).

To analyze the effects of intrinsic flexibility in the mechanical structure of the arm, a multi-body simulation was set up in the commercial simulation tool SIMPACK®, featuring free-floating target and chaser satellites, as well as a flexible model of the robotic manipulator. The tool enables the assesment of the structural eigenfrequencies, as well as the tracking error induced by the flexibility when commanding representative joint trajectories. This model concentrated on link flexibility. Figure 7, **Bottom** shows the flexible links, connecting the joints, that were modeled as Timoshenko beams. The arm segments which enclose the joint drives and the gripper were modeled as rigid.

The results of the analysis for the docking maneuver from capture to clamping position, in which the loads on the arm are most prominent, are summarized in Table 2. Furthermore, Figure 7, **Top** depicts the time response for a 60 s duration of the maneuver. In this case, the maximum displacement due to flexibility is Δ_*max*_ = 3.6 mm. This displacement is mainly caused by the acceleration profile of the commanded trajectory. After the maneuver ends, a residual vibration Δ_*f*_ = 0.2 mm, with a frequency of 0.31 Hz, can be observed in Figure 7, **Top**. Δ_*f*_ is relevant for the calculation of the required accuracy of the clamping interface. Note that in order to minimize flexibility effects, the velocity of the commanded arm trajectory can be reduced. As can be seen in Table 2, by doubling the maneuver time to 120 s, the error is decreased significantly, from 3.6 to 0.9 mm. This analysis shows that the effects of the link flexibility on the end-effector position are well within the clamping mechanism structural design requirements.

**Table 2:** Structural parameters for the flexibility simulation: outer and inner diameter of the cylindrical tube (*d*_*i, o*_), Youngs modulus (*E*), and Poisson's ratio (ν).

**Structural parameter**	***d***_***i***_ **[mm]**	***d***_***o***_ **[mm]**	**E [GPa]**	**ν [-]**
	123	127	69	0.334
	***f*_1_(*t*_0_) [Hz]**	***f*_1_(*t*_*f*_) [Hz]**	**Δ_*max*_ [mm]**	**Δ_*f*_ [mm]**
Berthing (60 s)	0.29	0.31	3.6	0.2
Berthing (120 s)	0.29	0.31	0.9	n.d.

*Results of the flexibility analysis for berthing maneuver: f_1_(t_0_) first eigenfrequency at maneuver start, f_1_(t_f_) first eigenfrequency at maneuver end, Δ_max_ maximum flexible displacement during maneuver, Δ_f_ amplitude of the residual vibration after the maneuver end*.

### 5.3. Joint loads

In order to verify the capability of the robotic manipulator, it is necessary to analyze the loads in the robot joints and in the robot gripper throughout the different phases of the mission. We recall the phases here for convenience: the approach, the capture, the rigidization, the de-tumbling, and finally, the repositioning of the chaser onto the LAR for the subsequent de-orbiting maneuver. The first phase has negligible loads in comparison to those in which the target is attached to the gripper. During the capture phase, forces arising from unexpected impacts with the target could act on the robot. However, thanks to the impedance control of the robot joints, it is assumed that any resulting impact will be of limited magnitude, given an appropriate tuning of the control gains (Uyama et al., 2012; Rodriguez Perez et al., 2018). The remaining three phases instead, present the highest risk for exceeding the internal torque limits of the joints, as defined in section 4.2, although the final repositioning phase can, in fact, be suitably timed to ensure its feasibility. We will concentrate here on the tumbling phase following the grasping.

We now analyze the robot internal forces during the tumbling motion, after the rigidization. Note that the robot needs to provide the necessary internal structural forces in order to keep the chaser in its position relative to ENVISAT, given that the chaser GNC is assumed to be switched off. Due to the tumbling motion, the chaser will experience centrifugal and tangential forces, which are a function of the tumbling rate of the compound and of its position with respect to the compound center of mass. Of interest is the moment at the robot end-effector, which represents the point in the robot structure with the highest load resulting from the apparent forces, due to the greatest moment arm from the point of application. This is equivalent to the moment in which the last joint of the robot has to apply, which is dependent on the choice of the arm delivery point position. The latter is also strongly conditioned by the requirements of the chaser GNC system, in order to guarantee collision avoidance with the target and to guarantee visibility to the proximity sensor.

**Figure 8:**
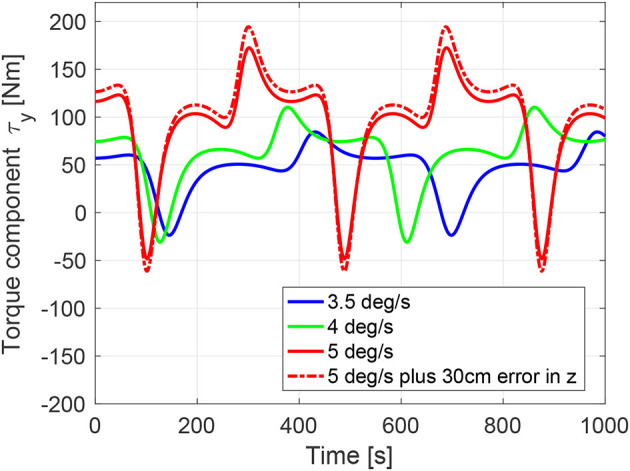
Gripper torque y-component during post-grasping tumbling motion. Simulation results shown for three target initial angular velocities (about y-component only) and for an additional 30 cm positioning error (+ve z-axis) of the chaser with respect to the predefined arm delivery point.

By considering the worst case scenario as defined by ESA, in which ENVISAT initially spins about its body-fixed y-axis (unstable, since the intermediate axis of inertia), the robot internal torque at the end-effector is now determined. The sum of the centrifugal and tangential forces acting at the chaser centre of mass is given as

(1) $$F_{c}=m_{c}\left({\dot{\omega }}_{t}\times r_{c}+\omega_{t}\times \omega_{t}\times r_{c}\right),$$

where *m*_*c*_ and **r**_*c*_ are the chaser's mass and centre of mass position with respect to ENVISAT's centre of mass (we neglect the mass of the chaser, for simplicity), respectively. Furthermore, **ω**_*t*_ and ${\overset{{}^{\circ}}{\omega }}_{t}$ are ENVISAT's angular velocity and acceleration, respectively. Given the position of the robot end-effector as **r**_*g*_, the moment at the end-effector is then given as

(2) $$\tau_{g}=\left(r_{c}-r_{g}\right)\times F_{c},$$

from which the nonlinear dependence of **τ**_*g*_ on **r**_*c*_ becomes evident. Typical profiles of the y-component of **τ**_*g*_ are shown in Figure 8, **Left**, having assumed the following numerical values for the given constants: *m*_*c*_ = 1380.0*kg*, **r**_*c*_ = [2.6, 0.0, 3.6]*m*, **r**_*g*_ = [3.9, 0.7, 1.0]*m*, as well as an ENVISAT inertia given as [17023.3 397.1 -2171.4; 397.1 124825.7 344.2; -2171.4 344.2 129112.2] kg *m*^2^. A rotation of 35 degrees is also introduced, to account for the position of the grasping point on the adapter ring (*y*-coordinate of **r**_*g*_ = 0.7*m*). A numerical analysis revealed that the chosen value of the *x*-component of **r**_*g*_ minimizes the torque values of interest.

From the plot we can deduce that the joint limits are reached for the worst case scenario of 5*deg*/*s*, recalling the value of the maximum joint peak torque of 176 Nm. The dotted red line shows the case in which a 30 cm positioning error of the chaser in the body-frame z-axis occurs. We observe a maximum value of 195 Nm. Note that for lower initial angular rates the torques are acceptable, as shown by the green and red curves in the plot, for 4 and 3.5*deg*/*s* initial angular velocity respectively, for which the maximum internal torques are below 85–110 Nm. Note also, that the case of target initial angular velocity about the body-fixed z-axis (stable, since maximum axis of inertia), yield torque values below 80 Nm.

## 6. System control design, simulations and analyses

In this section, elements of the control system are described and analyzed. In particular, the image processing is looked at for both the robotic capture and the chaser repositioning phases. Following, the coupled controller for the robot-navigation system is addressed.

### 6.1. Image processing

Both during grasping and fixation tasks, the image processing algorithm estimates the relative roto-translation (6 degrees of freedom pose parameters) from the camera to the target. The two tasks involve a different camera mounting, as well as different viewing points. In particular, the camera mounted on the robot end-effector observes the grasping point on the LAR during the whole approach maneuver, while the cameras for the fixation task are mounted on the chaser satellite in order to observe the LAR during the fixation maneuver. In this regard, the performance of the visual tracking is very important, as it is the most important contributor to the positioning accuracy of the robotic arm and therefore, also defines the worst case positioning error in translation and rotation as design drivers for both, the gripper and the clamping mechanism.

**Figure 9:**
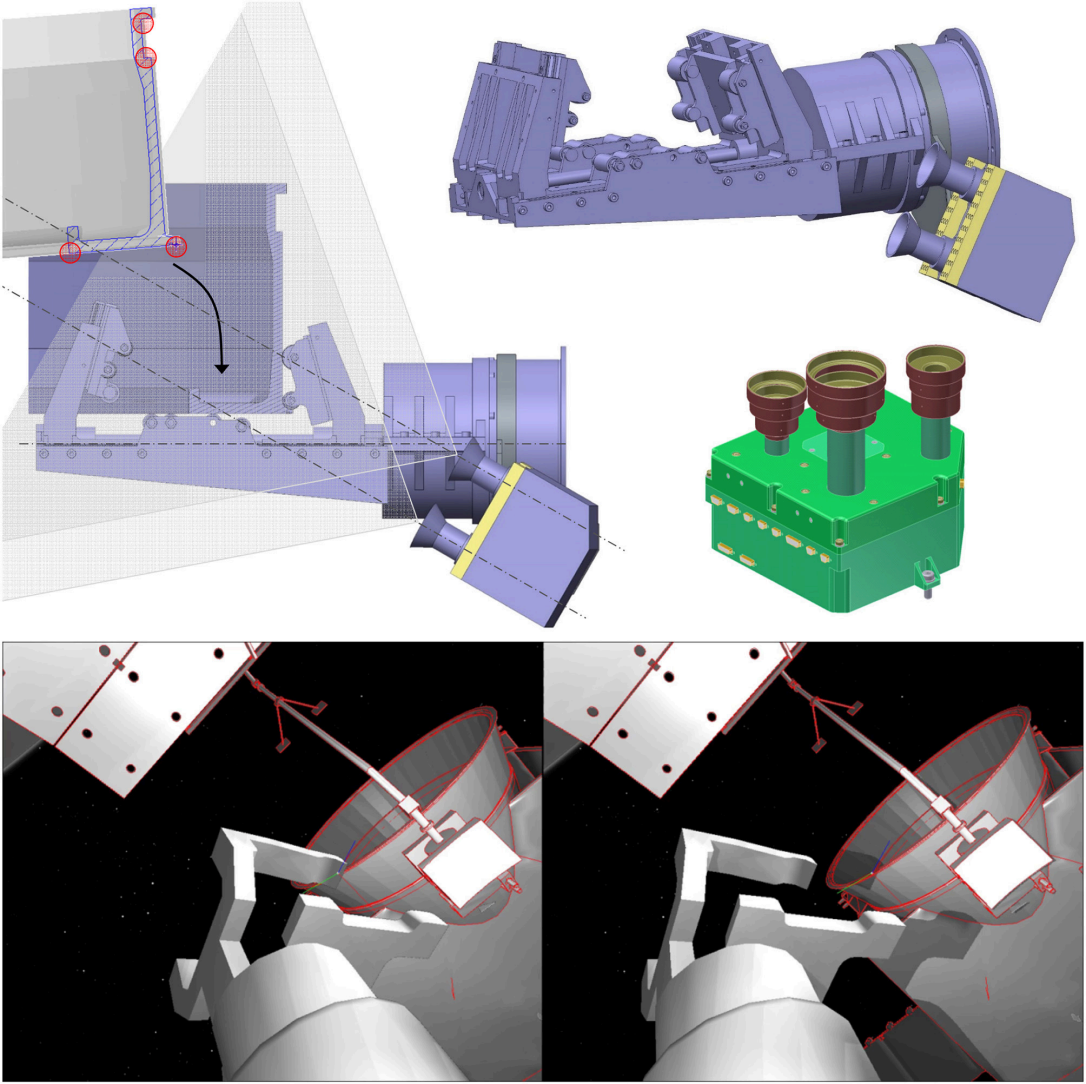
Arm stereo camera system with illumination units mounted on the gripper with mounting bracket **(Left and Top Right)** and platform-mounted camera system for relative spacecraft pose and motion estimation **(Middle Right)**. Example fo end-effector camera image rendered with ASTOS **(Bottom)**.

The robotic manipulator features a stereo camera system mounted laterally on its end-effector, including an integrated illumination system, shown in Figure 9, **Top**. The camera is panned and tilted, such that it allows continuous observation of the grasping point throughout the approach phase. A platform-mounted camera system with a vision-based sensor for rendezvous (VBS) is shown. While a space-proven LIDAR is designed for the chaser rendezvous and robot arm approaches, the VBS is envisaged to act as a visual sensor for positioning the chaser next to the adapter ring prior to closing the clamping mechanism. Using the same image processing algorithm as for the robot arm camera system, the VBS is conceived for target model building, matching as well as relative pose and motion estimation during this phase (Oumer et al., 2015). The system is heritage from the VIBANASS (Kaiser et al., 2013) project and comprises three cameras with different focal lengths for far-, mid- and close-range relative navigation.

Using these images, a visual tracking algorithm (Panin, 2011) detects the visible adapter ring edges and matches them to an internal model. The measured tracking error between the cameras and the grasping point on the LAR is the input to a visual servo, which is necessary for achieving an accurate relative positioning of the gripper. The main processing flow starts with a predicted pose that must be initially provided by means of robot kinematics and trajectory planning. This prediction is refined by minimizing a cost function (visual matching error), through a local optimization algorithm.

In particular, the optimization algorithm consists of a fast nonlinear least-squares minimization implemented in C++, and employs a simplified three-dimensional geometric model of the target, showing at least the relevant features (especially the shape of the grasping point), however with a reduced complexity with respect to the complete engineering model. The latter requirement is necessary to avoid overloading the system memory and computational power for real-time processing with a frequency of 10Hz. Such a model was prepared before testing. We remark that the stereo system is used as a multi-camera configuration so that the visual tracking should be able to proceed with a monocular camera, but slightly lowered accuracy. This configuration is used due to the required redundancy in space electronics to minimize the mission costs.

During pose estimation, the matching error is computed using the stereo camera images and projection parameters. This error measures the discrepancy between the projected geometry model, under a given pose hypothesis and the lines detected on each image, by using a contour sampling and matching technique. Residual errors and their derivatives with respect to the pose parameters (Jacobian matrix) are computed online, and used to update the pose in an iterative fashion, until convergence. Failure cases are also reported, in case the final matching error exceeds a safety threshold, or the estimated pose drifts too far away from the initial prediction.

For the mathematical formualtion of the visual tracking problem, consider a rigid body motion, given by the Euclidean group *SE*(3) based on Lie algebra

(3) $$T_{t}=\left[\begin{array}{cc} R & t\\ 0 & 1 \end{array}\right]$$

where *R* is a (3 × 3) rotation matrix and **t** a translation vector. A singularity-free parametrization around the current estimate, *T*_*t*_, is obtained by taking the tangent space *SE*(3) to the manifold at the last pose *T*_*t*−1_, given by an arbitrary vector *μ*_*t*_ at time t

(4) $$T_{t}=T_{t-1}\delta T(\mu_{t})$$

where the local incremental transform δ*T* is a singularity-free parametrization around *μ* = 0,.

Notice that *μ* = (**ω**, **v**) represents linear and angular motions in local coordinates of *T*_*t*−1_, and is defined through the exponential map

(5) $$\begin{array}{c} \sigma T=\exp(\overset{6}{\underset{i=}{\sum^{\text{​}}}}G_{i}\mu_{i}) \end{array}$$

where *G*_*i*_ are 4 × 4 basis generators. For clarity, time subscripts t and t-1 are omitted. The 3D model point **x** = [*x y z* 1]^*T*^, sampled along the circular nozzle rim, is expressed in homogeneous coordinates and projected onto a given camera **y** = [*u v*]^*T*^ by computing

(6) y=π(K·T·δT(μ)·x)

where the operator π() transforms from homogeneous to Euclidean 2D coordinates under perspective camera model and *K* is a projection matrix, obtained through camera calibration.

We seek to minimize the cost function

(7) $$\hat{\mu }=\arg\underset{\mu }{\min}\sum_{i}^{n_{p}}||e_{i}|{|}^{2}$$

(8) $$=\arg\underset{\mu }{\min}\sum_{i}^{n_{p}}||s_{i}-y_{i}(\mu )|{|}^{2}$$

to estimate the pose by local optimization method such as Gauss-Newton and Levenberg-Marquardt, where **e** is the residual, **s** is the image coordinates of the matching edge to the projected point **y**, and *n*_*p*_ is the number of matching pairs.

The orbital environment conditions were simulated with the ASTOS simulation tool. This allows uploading computer-aided design models of the robot and of ENVISAT, as well as positioning Sun and Earth in relation to the two satellites in any realistic fashion. As an example of a camera image rendered with ASTOS, is shown in Figure 9, **Bottom**.

The trajectory of the cameras relative to the target was provided by the motion planner, as described in Lampariello and Hirzinger (2013). The main requirement for the motion planning task was that the grasping point and sufficient features of the LAR and of the target satellite are visible throughout the complete motion. Furthermore, a requirement was posed on the velocity of the cameras with respect to the target, as it was found that high velocities lead to a loss of convergence of the image processing.

**Figure 10:**
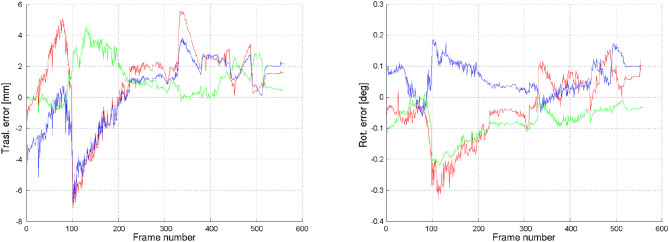
Error in pose estimate (translation and rotation) for an approach maneuver of the robot end-effector to the LAR.

Using this environment, a Monte-Carlo analysis was conducted, which purpose was to: (1) get an assessment of the expected pose estimation error, and (2) specify for which lighting conditions the proposed method works to a sufficient degree. The analysis was performed for the arm approach phase to the dedicated grasp point. In total, 120 sequences were used, related to two different tumbling states of ENVISAT (defined by the initial angular velocity of 5*deg*/*s* around the y and z-axis, respectively), 10 different start times for the robot approach maneuver (this guarantees that each of the ten sequences start with different initial orientations with respect the given Sun position and as such different illumination conditions), and 6 sunlight directions (for the three Cardinal directions, in the positive or in the negative sense). Each sequence consisted of 560 frames, sampled at 1/10 s. per frame (hence, 56 s. approach time). The main reference system was centered about the Earth, and the two satellites were located at a given altitude (about 700 km) along the +x axis. The sunlight directions were also expressed in this reference system. Typical error sequences are outlined in Figure 10. The results of the Monte-Carlo analysis are however more revealing as shown in Table 3.

**Table 3:** Monte-Carlo average error results with rows indicating tumbling rotation axis and approach start time (seq. 1–10), columns referencing sunlight direction and each cell containing the error for rotation and translation in the form [deg, mm].

**Tumbling sequence**	**Sun −x**	**Sun +x**	**Sun −y**	**Sun +y**	**Sun −z**	**Sun +z**
y-axis, seq. 1	0.42, 9.55	0.16, 3.80	0.16, 3.82	0.16, 3.87	0.35, 8.88	0.16, 3.78
y-axis, seq. 2	0.42, 9.47	0.14, 3.59	0.15, 3.59	0.16, 3.64	0.17, 3.62	0.15, 3.57
y-axis, seq. 3	0.37, 9.81	0.12, 3.30	0.14, 3.92	0.14, 4.00	0.14, 3.75	0.35, 10.68
y-axis, seq. 4	0.40, 10.13	0.14, 3.19	0.16, 3.38	0.16, 3.36	0.15, 3.36	0.15, 5.07
y-axis, seq. 5	0.39, 11.53	0.15, 3.29	0.16, 3.40	0.37, 10.56	0.16, 3.45	0.20, 5.02
y-axis, seq. 6	0.43, 10.15	0.15, 3.35	0.15, 3.30	0.16, 4.94	0.17, 3.57	0.15, 3.41
y-axis, seq. 7	0.38, 9.35	0.15, 3.69	0.15, 3.31	0.22, 5.37	0.12, 4.14	0.16, 2.86
y-axis, seq. 8	0.16, 3.41	0.39, 11.14	0.17, 3.81	0.15, 4.17	0.30, 9.37	0.17, 3.91
y-axis, seq. 9	0.15, 3.39	0.13, 4.23	0.15, 3.52	0.13, 3.21	0.22, 4.92	0.15, 3.49
y-axis, seq. 10	0.15, 3.21	0.16, 4.23	0.16, 3.34	0.15, 3.27	0.16, 3.24	0.14, 3.90
z-axis, seq. 1	0.19, 4.81	0.18, 4.92	0.17, 4.78	0.12, 4.85	0.14, 4.49	0.18, 4.76
z-axis, seq. 2	0.15, 3.48	0.17, 3.49	0.16, 3.39	0.15, 3.44	0.16, 4.85	0.16, 3.33
z-axis, seq. 3	0.14, 2.97	0.39, 10.93	0.14, 3.53	0.14, 3.46	0.15, 4.22	0.14, 3.46
z-axis, seq. 4	0.16, 3.33	0.41, 10.43	0.16, 3.30	0.14, 3.22	0.15, 4.17	0.16, 3.28
z-axis, seq. 5	0.17, 3.33	0.21, 4.86	0.16, 3.36	0.18, 3.79	0.14, 4.10	0.16, 3.21
z-axis, seq. 6	0.14, 3.87	0.16, 3.38	0.16, 3.31	0.18, 4.47	0.12, 3.95	0.16, 3.33
z-axis, seq. 7	0.16, 4.57	0.14, 3.48	0.16, 3.83	0.22, 5.46	0.12, 4.31	0.15, 3.76
z-axis, seq. 8	0.21, 4.98	0.16, 4.63	0.16, 4.59	0.17, 4.89	0.12, 4.02	0.16, 4.40
z-axis, seq. 9	0.19, 4.73	0.18, 4.55	0.18, 3.99	0.19, 4.53	0.17, 4.51	0.18, 3.90
z-axis, seq. 10	0.14, 2.91	0.15, 3.39	0.14, 3.53	0.14, 3.46	0.15, 4.14	0.14, 3.47

Each entry in the table is related to an approach sequence to the tumbling client. The rows indicate the tumbling rotation axis (y/z) and approach start times (seq. 1,…,10), while the columns reference the sunlight direction. Each cell reports the average errors of rotation and translation [deg, mm], respectively, given by the magnitudes of rotation and translation vectors. These results were obtained after tuning of the image processing algorithm parameters. They are as such optimal results, from the point of view of the proposed method. Generally, it was found that a lack of light contrast in critical areas does not allow detecting some of the important lines of the simplified model for the purpose of the tracking, which in turn does not allow accurate pose estimation. In other words, the estimator remains trapped in local minima due to insufficient measurements of the 3D model position. As a result, it can be concluded that the proposed method works in the majority of cases, but not for all orientations of ENVISAT with respect to the Sun during its tumbling motion. It can also be concluded that it is difficult to define ideal positions of the Sun for ideal lighting conditions. This is because in each column there is at least one sub-sequence with significant errors, with the exception of the third column (Sun in -y). No correlation can be seen between the pose estimation error and the direction of the sunlight. It was also recognized that the LAR provides a particularly difficult pose estimation task, due to its lack of evident and easily recognizable features. The worst case results for the pose estimate error turned out to be ±2.5 cm. Further efforts to increase the robustness of the image processing will include introducing a third camera with a different perspective, e.g., on the chaser satellite.

### 6.2. Robotic arm - GNC coupled control

Manipulator operations were developed in accordance with the given inter-dependencies between the arm controller and the chaser's guidance, navigation and control (GNC) subsystem. At the arm delivery point, the GNC stabilizes the free-flying base in closed-loop with the relative pose estimation system, while the arm approaches the dedicated grasp point. During the robot arm approach to the grasping point on the LAR, the arm movement introduces disturbance forces and torques on the stabilized base. On the other hand, the thrusters action to control the base can impact on the arm end effector positioning accuracy.

The strategy adopted for the control of the manipulator and the GNC is summarized in this section. Usually, torque-based controllers are employed when the manipulator interacts with objects, especially when the latter is a free-floating target satellite (Artigas et al., 2015). Therefore the control law will be based on an impedance behavior between the robot end effector and the target point, i.e., the LAR. Let us introduce the general equation of motion for a space robot as Yoshida (2000):

(9) $$\left[\begin{array}{cc} H_{b} & H_{bm}\\ H_{bm}^{T} & H_{m} \end{array}\right]\left[\begin{array}{c} {\overset{..}{x}}_{\text{b}}\\ \overset{..}{q} \end{array}\right]+\left[\begin{array}{c} c_{b}\\ c_{m} \end{array}\right]=\left[\begin{array}{c} F_{b}\\ \tau \end{array}\right],$$

where ${\text{H}}_{b}\in {\mathbb{R}}^{6\times 6}$, ${\text{H}}_{m}\in {\mathbb{R}}^{7\times 7}$, ${\text{H}}_{bm}\in {\mathbb{R}}^{6\times 7}$ are the inertia matrices of the whole system, manipulator and the coupling between the base and the manipulator, respectively. The vectors ${\ddot{\text{x}}}_{b}\in {\mathbb{R}}^{6\times 1}$ and $\ddot{\text{q}}\in {\mathbb{R}}^{7\times 1}$ are the acceleration of the base and the acceleration of the robot joints; ${\text{c}}_{b}\in {\mathbb{R}}^{6\times 1}$ and ${\text{c}}_{m}\in {\mathbb{R}}^{7\times 1}$ are the non-linear velocity dependent terms on the base and on the manipulator, respectively. ${\text{F}}_{b}\in {\mathbb{R}}^{6\times 1}$ is the force-torque vector acting on the center of mass of the base-body and **τ**∈ℝ^7 × 1^ is the internal torque vector. The kinematics between the operational space and the joint space is described as follows:

(10) $${\dot{x}}_{e}=J_{b}{\dot{x}}_{b}+J_{m}\dot{q},$$

where ${\overset{{}^{\circ}}{\text{x}}}_{\text{e}}\in {\mathbb{R}}^{6\times 1}$ is the end effector velocity vector, ${\text{J}}_{b}\in {\mathbb{R}}^{6\times 6}$ and ${\text{J}}_{m}\in {\mathbb{R}}^{6\times 7}$ are the Jacobian matrices of the base and manipulator, respectively.

The robotic arm considered in e.deorbit is a redundant robot. The redundancy enables a motion in the nullspace (Siciliano et al., 2009) which has to be taken into account in the control law. The requirements of the manipulator controller are twofold. First, the task of tracking the LAR on Envisat should be fulfilled in Cartesian space with a compliance behavior; secondly, the motion in the null space of the robot should be controlled. The mentioned above requirements can be achieved with the following control law:

(11) $$\tau =\underset{\tau_{c}}{\underset{︸}{J_{g}^{T}F}}+\underset{\tau_{n}}{\underset{︸}{(I-J_{g}^{T}{\bar{J_{g}}}^{T})\Gamma }},$$

where **τ**∈ℝ^7 × 1^ are the input torques to the manipulator. Notice that $J_{g}\in {\mathbb{R}}^{6\times 7}$ is the generalized Jacobian matrix which is defined as follows:

(12) $$J_{g}=J_{m}-J_{b}H_{b}{}^{-1}H_{bm}.$$

The generalized Jacobian in (12) has not a square structure, therefore the dynamically consistent generalized inverse $\bar{J_{g}}\in {\mathbb{R}}^{7\times 6}$ has been exploited in (11) and it is defined as: $\bar{J_{g}}=H_{g}^{-1}J_{g}^{T}\Lambda$, where $H_{g}\in {\mathbb{R}}^{7\times 7}$ is the generalized inertia matrix and **Λ**∈ℝ^6 × 6^ is the inertia matrix in Cartesian space (Umetani and Yoshida, 1987).

The designed control law in (11) is composed by two terms which allow fulfilling the control requirements. In particular, **τ**_**c**_ is the torque contribution which controls the end-effector. This is the function of a Cartesian virtual force **F**, later defined, which allows compliance between the end effector and the grasping point. **τ**_**n**_ is the torque contribution which controls the null space motion with a generalized joint torque vector **Γ**. This is defined as $\Gamma =-D_{n}\dot{q}$ where $D_{n}\in {\mathbb{R}}^{7\times 7}$ is a damping matrix. Notice that **Γ** acts as an internal damping torque and it will not interfere with the end effector motion.

The compliance during the approaching phase is provided with the virtual Cartesian forces vector **F** at the end-effector in (11) and it is modeled like a PD (proportional-derivative) behavior. Therefore, **F** is defined as:

(13) $$F=K_{P}\Delta x+K_{D}\Delta \dot{x}.$$

The matrices **K_P_** and $K_{D}\in {\mathbb{R}}^{6\times 6}$ are positive definite and they represent the stiffness and damping gains of the controller. The vectors Δ**x**, $\Delta \overset{{}^{\circ}}{\text{x}}$ are the position and velocity error vector respectively. Notice that these vectors describe the error in translation and orientation expressed as in De Stefano et al. (2015).

Equation (11) is then computed as an internal joint torques to the free-floating robot dynamic (9).

During the approach phase of the robot to the grasping point, the arm movement introduces disturbance forces and torques on the base. The control of the base and the manipulator can be performed applying two main strategies: combined control (De Stefano et al., 2018) or a coupled control strategy (Telaar et al., 2017b). In this work the coupled control strategy has been considered as the interface of two systems, i.e., the base and the manipulator. Thus the GNC controls the attitude of the chaser with a frequency of 1 Hz and it operates relative to the target. The robot controller controls the end-effector position with a frequency of 1 KHz.

Both controllers will exchange data. Position and orientation data between the chaser and the target are provided by the chaser to the robot controller. On the other hand, the robot controller will exchange data with the chaser by means of forces and torque computed at the robot base, calculated as:

(14) $$F_{rb}=-H_{bm}\overset{..}{q}-c_{b}.$$

The GNC control is usually based on a PID (Proportional-Integral–Derivative) control computed at 1 Hz which receive as input the disturbance force due to the motion of the manipulator computed in (14) and provides as output the control force **F_b_**. More details about the GNC architecture can be found in Telaar et al. (2017a).

In order to better understand these inter-dependencies, a coupled control simulation environment was set up and analyzed in Simulink. The simulation featured free-floating dynamics of the two spacecraft, an arm controller with ideal pose estimates between the manipulator end-effector camera and the LAR, as well as the chaser GNC, provided as a black box by both industrial partners, respectively. While the GNC was assumed to operate at 1*Hz*, the arm control was assumed to run at 1*kHz*, thus neglecting the sampling time of the end-effector camera pose estimation sampling time (typically 10 Hz) for model simplification. Simulations consider an initial motion of Envisat of 5*deg*/*s* and multiple starting positions and orientations within and outside the GNC error box of 10 cm and 0.5*deg* relative positioning accuracy. The aim is to analyze the accuracy of the gripper positioning with respect to the grasping point, while the GNC is actively stabilizing the relative pose of the two spacecraft. Further details can also be found in Telaar et al. (2017b) and in Telaar et al. (2017a).

**Figure 11:**
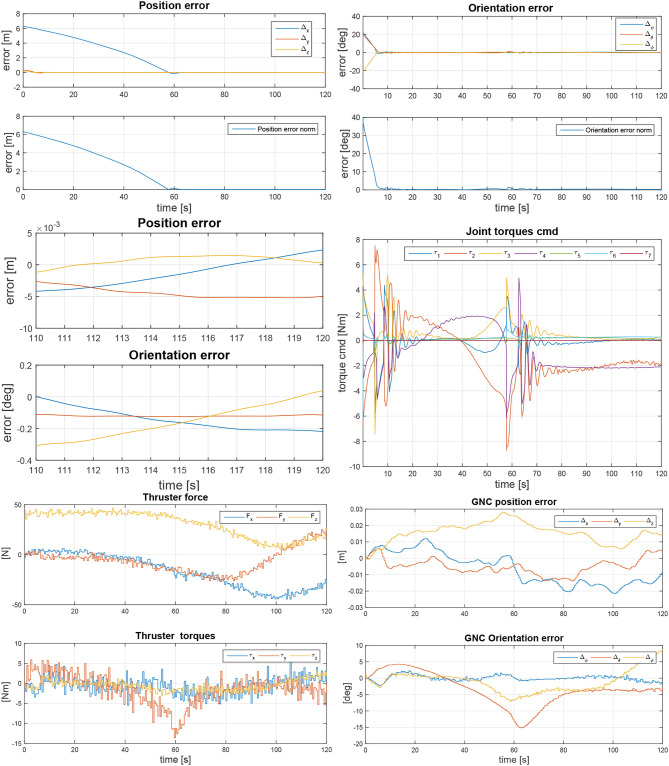
Arm end-effector positioning and orientation error with respect to grasp point for a target satellite rotation reference scenario of 5*deg*/*s* around y-axis **(Top)**. Zoom-in at final arm end-effector positioning and orientation error with respect to grasp point **(Middle, Left)** and commanded robot joint torques **(Middle, Right)**. GNC thruster forces and torques **(Bottom, Left)** and resulting GNC error **(Bottom, Right)**.

The gains of the controller in (13) are set as follows: **K_P_** = *diag*(350, 350, 350, 50, 50, 50), **K_D_** = *diag*(25, 25, 25, 15, 15, 15) and for the null space motion the damping matrix is **D_n_** = *diag*(2, 2, 2, 2, 2, 2, 2). Notice that the manipulator joints are considered to be ideal and therefore no model of friction is considered. Figure 11, **Top** shows the arm end-effector position and orientation error with respect to the LAR, both in components and in norm, respectively. Figure 11, **Middle, Right** depicts on the right-hand side the commanded robot joint torques of the controller in (11). The thruster activity is shown in Figure 11, **Bottom, Left**, indicating the generated forces and torques. Due to the fact that the chaser must follow the tumbling target, the thrusters are highly active over time. The resulting GNC error is plotted in Figure 11, **Bottom, Right**.

Next to tool center point (TCP) positioning accuracy, the effect of active thrusting on the end effector position was of special interest. Figure 11, **Middle, Left** for example shows a zoom-in view of the final arm approach while Envisat is tumbling on its y-axis. The simulation automatically finalizes once the position error below 4 mm and 0.3*deg* is reached. For all tested stack rotation options and starting conditions, the final grasp position could always be reached within this defined error box. Looking at the zoom-in plot of the last 10*s* of the simulation, it becomes obvious that the GNC thrusting in Figure 11, **Bottom, Left**, only has a minor impact on the arm end-effector position. This is due to the very low frequency of the actuation, high inertia of the satellite and the higher visual servoing estimation rate. This is also due to the slow arm movement that was used here, as the arm positioning accuracy was focused on. Overall it can be stated that the coupled control approach it suitable to solve the described free-flying manipulator task. Both GNC thrusting and arm movement interaction forces and torques have only minor effect on the respective counterpart. Control system stability here was shown with some numerical examples. However, the rigorous mathematical proof of stability of a further detailed coupled controller is currently ongoing research and therefore intended as future work.

## 7. Conclusion and future work

In this paper we presented the robotic design and operational strategy developed for capturing ENVISAT in the scope of the e.deorbit phase A and phase B1 studies. The redundant mechatronic design of a torque-controlled robot arm was presented, which allows for an impedance-based grasping strategy, minimizing the effect of unexpected impacts during capture. Novel, mission-specific designs were presented for the robot arm gripper and for a clamping mechanism, which is necessary for securing the chaser satellite onto ENVISAT for performing the final deorbiting maneuver. Both the gripper and the clamping mechanism were designed to achieve full form-closure with the launch adapter ring of ENVISAT.

The arm kinematics were validated using the method of the capability map. Dynamic simulation analyses showed that the effect of robot arm link flexibility on the gripper position is neglegible. The analysis of the loads in the robot joints during some of the most critical phases of the mission showed that the robot design is suitable for a wide range of the selected operational scenarios. The outcome of a Monte Carlo analysis of the visual tracking algorithm used to provide pose estimates of the target was on average satisfactory. Finally, the coupled control approach between robotic arm and GNC was shown to work robustly within the given simplifying assumptions, yielding sufficient pointing accuracy and showing only minor interacting disturbance effects between the chaser and the robot arm controllers.

Future work will focus on the implementation and validation of a visual servo. The validation method presented here, based on simulation, will be extended with experiments on DLR's OOS-SIM robotic facility (Artigas et al., 2015). Furthermore, the coupled control method will be implemented in simulation to analyze the complete capturing and rigidization phases.

## Author contributions

SJ and RL led the described research and wrote most of the manuscript. The following authors conducted the subsequently named research and contributed the respective parts to the manuscript. MD and WR coupled control model. AG arm flexibility. OP capability map analyses. MP, QM, and MT gripper. NO visual servoing. BB arm kinematics and visualization. JR clamping mechanism. MP and SE headed the industrial collaboration on OHB and Airbus side, respectively, RB heads the e.deorbit mission at esa, and AA-S heads the robotics institute at DLR, of which all proofread the manuscript.

### Conflict of interest statement

The authors declare that the research was conducted in the absence of any commercial or financial relationships that could be construed as a potential conflict of interest.
